# Recombinant *Lactococcus lactis *can make the difference in antigen-specific immune tolerance induction, the Type 1 Diabetes case

**DOI:** 10.1186/1475-2859-13-S1-S11

**Published:** 2014-08-29

**Authors:** Sofie Robert, Lothar Steidler

**Affiliations:** 1 Clinical and Experimental Endocrinology (CEE), KU Leuven, 3000, Leuven, Belgium; 2 ActoGeniX NV, 9052, Zwijnaarde, Belgium

## Abstract

Especially in western civilizations, immune diseases that are driven by innocuous (auto- or allo-) antigens are gradually evolving to become pandemic threats. A particularly poignant example is type 1 diabetes, where young children are confronted with the perspective and consequences of total pancreatic β-cell destruction. Along these disquieting observations we find ourselves equipped with impressively accumulating molecular immunological knowledge on the ins and outs of these pathologies. Often, however, it is difficult to translate this wealth into efficacious medicines. The molecular understanding, the concept of oral tolerance induction, the benefit of using recombinant *Lactococcus lactis *therein and recent openings towards their clinical use may well enable turning all colors to their appropriate fields on this Rubik's cube.

## Antigen-specific immune therapy and recombinant *Lactococcus*, what, when and why

(Auto-) immune diseases are characterized by aberrant immune reactions of the immune system towards a limited number of normally innocuous foreign antigens (Ags) as is the case for allergies and asthma or self-Ags in autoimmune diseases. More than 80 different autoimmune diseases have currently been identified. These can target only one organ, such as in type 1 diabetes (T1D), or multiple organs, such as in rheumatoid arthritis. Autoimmune diseases affect around 7% of the western population and this collective prevalence is increasing alarmingly [1,2]. A similar observation is seen for allergic diseases where the prevalence of 20% in children of developed countries also slowly rises [3-5]. The underlying events triggering this excessive reactivity remain unknown, but clearly involve genetic predisposition, allowing inapt interaction of environmental factors with the immune system. The general consensus to treat allergy and autoimmune diseases is altogether unsatisfactory. The field mostly relies on non-specific systemic immune suppression or symptomatic treatment which unfortunately does not address the underlying Ag-specific reactivity and is usually associated with severe systemic side effects. This can be overcome with Ag-specific therapeutic strategies targeting only the excessive immune reactivity. In this review we focus on Ag-specific tolerance induction in general and develop T1D as a case study for Ag-driven (auto-) immune diseases, to introduce Ag-specific tolerance restoration by oral administration of recombinant *Lactococcus lactis *(*L. lactis*) bacteria. This innovative Ag-specific strategy for oral tolerance induction could open up new therapeutic possibilities in the emerging field of (auto-) immune diseases. Moreover, it points to a clear opportunity where recombinant *L. lactis *could make the difference in medicine.

*L. lactis' *genetic engineering and the finding that it can be used for therapeutic protein delivery was developed during the previous century. The crux of the idea is that *L. lactis *can constitutively produce functional, eukaryote derived, proteins, without any apparent major negative effect on its growth and physiology, even when present in the mucosa of mammalians. The basics thereof are robust and sound and have changed little, as can be judged by a wealth of literature [6-14], all using the same basic principles. Now is an exciting time to find that the field has evolved in such a way that it has become a clinical option now considered by physicians [15]. The field is ready to address real-life demands and standards should be set along the existing standard of care. The aim of this review is to provide such benchmarking and to show that a viable clinical development path is realistic. Below we will elaborate on T1D as a genuine therapeutic need, review current treatment options, how Ag-specific therapies were conceptually molded and touch upon the possibilities and downsides of using oral tolerance induction. We will further develop our rationale by pointing out that recombinant *L. lactis *may well be the ideal tools to bring Ag-specific oral tolerance induction to reality, with focus on our recent findings in treating animal models of T1D. Finally, we will give a general overview of our accomplishments in clearing the clinical path for recombinant *L. lactis*.

## Type 1 diabetes

**Figure 1:**
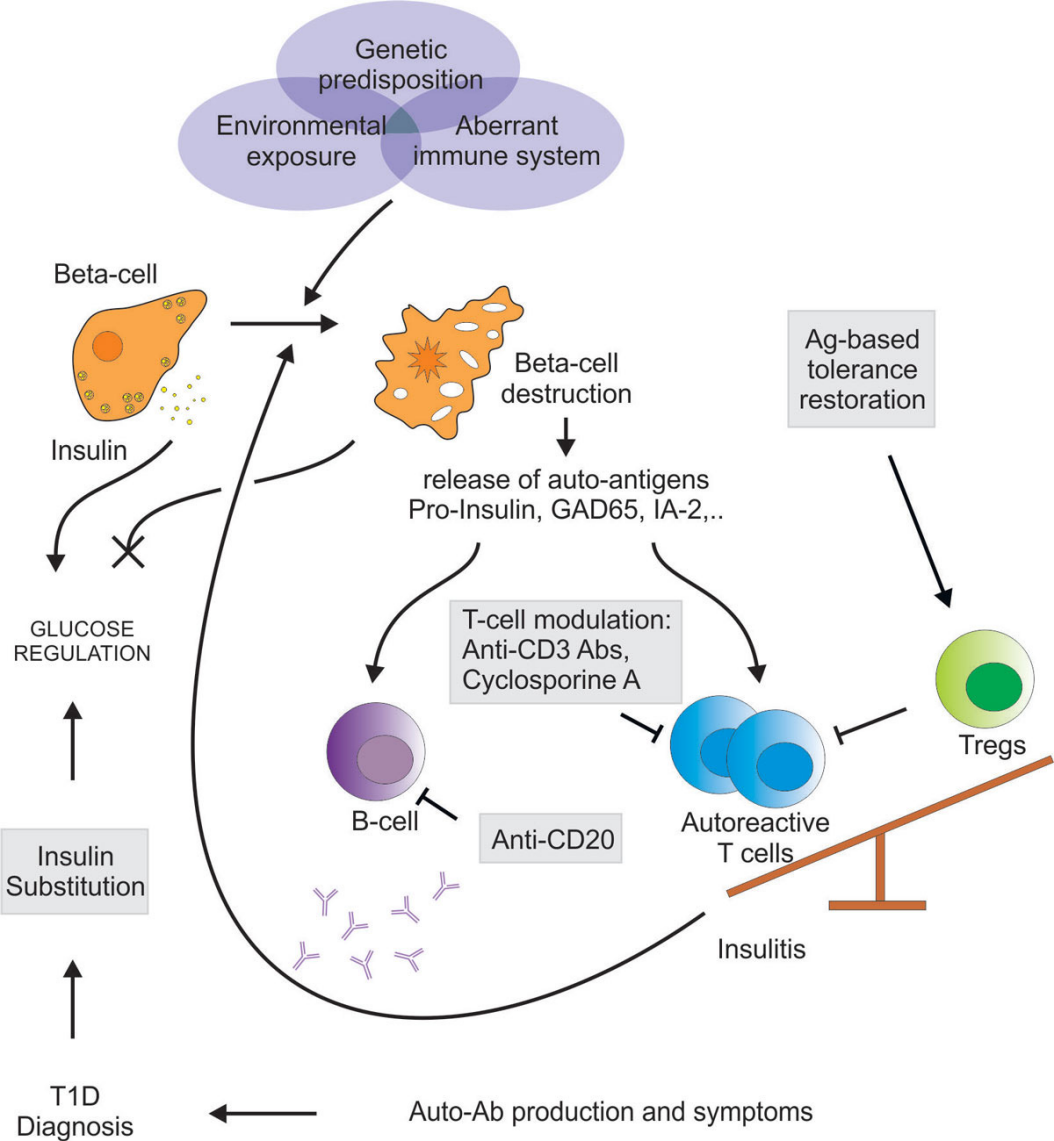
**T1D onset and development**. Genetic predisposition and environmental exposure interact with an impaired immune system to break tolerance towards pancreatic insulin-producing β-cells. Initial β-cell destruction leads to release of β-cell-specific auto-Ags, such as pro-insulin, GAD65, IA-2 and others. Upon auto-Ag-recognition, auto-reactive T cells expand, migrate to the pancreas (insulitis) and induce progressive β-cell destruction. In healthy individuals these auto-reactive T cells are controlled by regulatory T cells (Tregs). However, in T1D patients this regulatory counterpart is impaired causing a significant imbalance of this immune control mechanism. In addition, auto-Ag-activated B lymphocytes produce β-cell-specific auto-Abs. When patients present with clinical symptoms caused by insufficient β-cell-mass and subsequent aberrant glucose regulation, this auto-Ab profile allows for specific diagnosis of immune-mediated T1D. In general, T1D patients can only rely on exogenous insulin substitutions to stabilize their glucose metabolism. Possible therapeutic targets involved in the pathology of T1D are highlighted (gray boxes). Both systemic immunomodulation, amongst others monoclonal anti-CD3 antibodies and cyclosporine A, and Ag-based approaches have been explored to restore tolerance.

T1D is an eminent example of a well-studied chronic autoimmune disease characterized by the selective destruction of insulin-producing pancreatic β-cells by pathogenic self-reactive CD4^+ ^and CD8^+ ^T cells. The prevalence of T1D is estimated to number up to more than 20 million patients worldwide with the incidence in children under the age of 5 to double between 2005 and 2020 [16,17]. With the help of several *in vivo *models, such as the biobreeding rat and the non-obese diabetic (NOD) mouse, both of which develop T1D spontaneously, more insight has been gained in the mechanism of the autoimmune cascade. Due to speculative triggers, pancreatic β-cell Ags become presented by Ag-presenting cells that have infiltrated pancreatic islets (Figure 1) [18,19]. This will activate and expand pathogenic islet Ag-specific T cell populations [19,20] and eventually the natural balance between auto-reactive T cells and their regulatory counterparts is disturbed, leading to tolerance breakdown and a progressive β-cell loss (Figure 1). The immanent diminished release of the glucose-regulating hormone insulin will lead to elevated blood glucose levels or hyperglycemia. As for most autoimmune diseases there is no specific cure for T1D. Patients can only rely on exogenous insulin substitutions to normalize glucose levels, which however do not prevent complications of the pathology, thereby making it difficult to pursue normal life quality and normal lifespan.

## Standard of care: systemic immune suppression, which comes at a price

So far, current therapeutic strategies applying systemic immunosuppression have given the best results in clinical studies. Systemic immunomodulators, such as cyclosporine A and monoclonal anti-CD3 antibodies, target the general immune system in order to neutralize the β-cell-specific autoimmune reactivity.

Cyclosporine A, a pioneer amongst immunosuppressive agents, blocks immunity at one of its most crucial steps. This polypeptide inhibits T cell activation by inhibiting autocrine IL-2 synthesis and thus cell proliferation. The drug has already demonstrated efficacy in enhancing renal transplantations and preventing rejections [21]. Several clinical studies in newly-diagnosed type 1 diabetic patients showed disease remission associated with enhanced preservation of β-cell function by cyclosporine treatment [22-24]. However diabetes reversal was never sustained and was accompanied with severe forms of nephrotoxicity [25-27].

Monoclonal anti-CD3 antibodies, targeting the T cell receptor (TCR), are a unique class of immunomodulators in that they interact with both pathogenic effector T cells and regulatory T cells (Tregs, T cells that sustain tolerance). This dual mechanism of action works in two phases. In a first phase, recently activated T cells are transiently depleted or made blind to Ag by shedding and internalization of the TCR/anti-CD3 complex [28,29]. In a second, long-term phase, anti-CD3 antibodies preserve and induce Tregs [30,31]. In recently-diagnosed (early) diabetic NOD mice a short-term low-dose course of anti-CD3 at a total dose of 25 µg could reverse diabetes in 80% of the treated mice [32,33]. These promising preclinical studies have led to construction of two humanized anti-CD3 antibodies, namely hOKT3y1 (Teplizumab) and ChAglyCD3 (Otelixizumab) and their clinical testing in newly-diagnosed type 1 diabetic patients [34,35]. Both drugs could preserve β-cell function for up to one year, after which the β-cell destruction gradually continued [36-38]. A follow-up study of Otelixizumab showed a significant preservation of β-cell function for up to 4 years post-treatment [39]. Increasing administered doses of Teplizumab with 40% did not ameliorate therapeutic efficacy and was associated with increased presentation of drug-related side effects, such as cytokine release, headaches, fever and rash [40]. In the Otelixizumab trial, several patients experienced a transient and self-limited reactivation of the Epstein-barr virus. However due to the positive therapeutic outcomes several phase III studies were conducted. The DEFEND-1 study, using Otelixizumab, did not meet its primary endpoint, mainly due to the unsubstantiated choice of validation parameters in addition to ad random choices made in dose reductions [41]. A second phase III study with Teplizumab (The Protégé study) also missed its primary endpoint. However, post-hoc analyses in subgroups of patients revealed better preservation of β-cell function when anti-CD3 was given in a full dose regimen, namely 17 mg administered at baseline and again after 6 months [42,43]. Similar observations of preserved insulin production with high-dose anti-CD3 were seen in the Autoimmunity-Blocking Antibody for Tolerance in Recently Diagnosed Type 1 Diabetes (AbATE) study [44]. Although the DEFEND-2, Protégé Encore and the SUBCUE trial were terminated, several investigator-led clinical studies with anti-CD3 are still being conducted. The At Risk-study is designed to evaluate whether teplizumab can help to prevent or delay disease onset in relatives at risk for T1D (http://ClinicalTrials.gov Identifier NCT01030861). The Delay-study evaluates whether Teplizumab will prevent the loss of insulin secretory capacity in individuals with recent, but not recent-onset, T1D (http://ClinicalTrials.gov Identifier NCT00378508). Also, a Phase I study on subcutaneous administration of Otelixizumab in T1D patients is ongoing (http://ClinicalTrials.gov Identifier NCT00946257). Taken together, these data still support anti-CD3 progress to full-scale phase III trials.

Although Ag-nonspecific systemic immune approaches have shown some therapeutic efficacy in the context of T1D - β-cell preservation, postponing the gradually decline in insulin production - one cannot but acknowledge that sustainable long-term effects could not be achieved. In addition, non-specific immune targeting provokes severe systemic side effects. Broad immune suppression renders the treated patient highly susceptible for pathogenic infections and may lead to the formation of malignancies. Notwithstanding the beneficial effects achieved with systemic immunomodulators, legitimate health risks remain associated with such therapeutic strategies. In conclusion it is fair to state that the shortcomings of current approaches limit access to effective therapies.

## Experimental Ag-specific T1D therapies are safe but show lack of efficacy

In most (auto-) immune diseases immune reactivity is restricted towards a limited number of (auto-) Ags. Auto-Ags targeted by auto-reactive T cells are continuously emerging and their disclosure relies heavily on the identification of autoantibodies present in patients or disease-predisposed individuals. Ag-specific control of these auto-reactive T cells remains the ultimate therapeutic approach, eliminating the risk of any unwanted systemic immune reaction.

Ag-specific approaches for (auto-) immune diseases require careful consideration. Introducing selected (auto-) Ags to a non-tolerogenic, already primed environment can boost (auto-)immune reactivity and aggravate disease. Correct choice of Ag, timing (window of opportunity), dose and frequency of administration are key aspects to take into consideration [45]. Accurate extrapolation of these parameters from preclinical animal, murine and porcine, models to man however remains a challenging task. Extrapolation should not just rely on mere body weight calculation but needs to take into account different physiological parameters, amongst others energy metabolism and renal function. Profound pharmacokinetic and pharmacodynamic studies in different animal models need to be carefully conducted to prevent unexpected events. Proper choice of adjuvants can favor a tolerogenic response. One example is the use of aluminum salts, which effectuates a Th2 response on top of enhanced Ag delivery and activation of Ag-presenting cells [46,47]. Moreover, the mode of administration remains crucial for tolerance inducing strategies.

Several pancreatic islet auto-Ags are involved in the pathogenesis of T1D, of which (pro-)insulin, glutamic acid decarboxylase 65 (GAD65) and tyrosine phosphatase-like protein ICA152 (IA-2) are the most prominent [48-50]. Parenteral, oral or nasal administration of said auto-Ags could prevent or delay T1D in the NOD mouse model underlining the prophylactic potential of Ag-specific therapies [51-55]. None of these preclinical studies were effective in established disease. So far, apart from a few exceptional cases dealing with cryptic epitopes, none of these preclinical studies have shown exacerbation of the autoimmune response, thereby highlighting their safe profile [56]. Hence, clinical translation has been disappointing. Multiple studies administering subcutaneous or intranasal insulin or GAD65 to newly-diagnosed T1D patients or to autoantibody-positive T1D-relatives did not improve the clinical outcome [57-62]. One study administering unprotected oral insulin induced a delayed onset of T1D, albeit only in a subgroup of diabetes-prone individuals with high baseline insulin autoantibodies [60]. Despite the disappointing absence of clinical efficacy, none of these clinical studies showed signs of disease aggravation, again reiterating the inherent safety of using these selected Ags.

## Oral tolerance induction: silver bullet, unmined field or the ever promise?

A number of studies point to the mucosal immune system of the gastrointestinal tract to induce Ag-specific suppression, relying on its privilege to induce tolerance to orally administered Ags. The lymphoid tissue of the gut covers more than 260 m² and contains one of the highest numbers of lymphocytes, rendering it the largest immune organ of the body. This mucosal immune system is excellently equipped to judge on unresponsiveness towards intestinal commensals and harmless food Ags and responsiveness towards pathogens. To maintain intestinal homeostasis, anti-inflammatory cytokines such as TGF-β and IL-10 are abundantly present in the gut and together with retinoic acid they create a highly tolerogenic environment [63]. In addition, excessive mucosal production and luminal secretion of IgA assists in neutralizing exotoxins and microbes. Sampling and presenting of luminal Ags is mediated by specialized M cells and dendritic cells, or even by epithelial cells through transcytosis. Gut Ag-presenting cells intrinsically favour the induction of Foxp3^+ ^Tregs in the gut-draining lymph nodes rather than activating effector T cells. Further, imprinting of gut-homing receptors (amongst others α4β7 and CCR9) guaranties the return of these Ag-specific Tregs to the gut [64]. Oral Ag administration makes use of these tolerogenic characteristics to suppress systemic Ag-specific reactivity. The underlying mechanism of oral tolerance induction depends on the dose of Ag administered, where, at least in preclinical models, low repetitive doses favour the induction of Tregs and high doses favour deletion or anergy of effector T cells [65]. Oral tolerance induction has successfully been applied in experimental autoimmune disease models such as collagen induced arthritis [66] and experimental autoimmune encephalomyelitis [67,68] and relates to the induction of Tregs in these experimental settings [69-71]. Suppressive cytokine production by Tregs (IL4, IL-10 and TGF-β) induces bystander suppression to other (auto-)Ags presented in the close vicinity. This spreading of regulatory responses can even make it redundant to provide the primary Ag, an advantage that can be used in the context of T1D where several auto-Ags are enrolled in the autoimmune cascade and the primary auto-Ag is not yet unambiguously identified.

Patients would definitely prefer the elegance of this safe and easy way of administration above the inconvenience of parenteral injections. The main challenge faced with oral tolerance induction is to efficiently deliver Ag doses of superior quality to the intestinal mucosa. Studies unveiled that, unless large quantities are used, intestinal passage causes Ag degradation before it can reach the mucosal immune system of the gut [72,73]. The cost of administering large quantities of high quality Ag being prohibitive makes that there is a strong need for adequate and effective vehicles to control oriented Ag-delivery to the gastrointestinal immune system. Moreover, if such vehicles could also deliver mucosal immune modulators such as IL-10, they could be pivotal in raising tolerogenic immune responses.

## *L. lactis*, a top-notch carrier for tolerance induction

**Figure 2:**
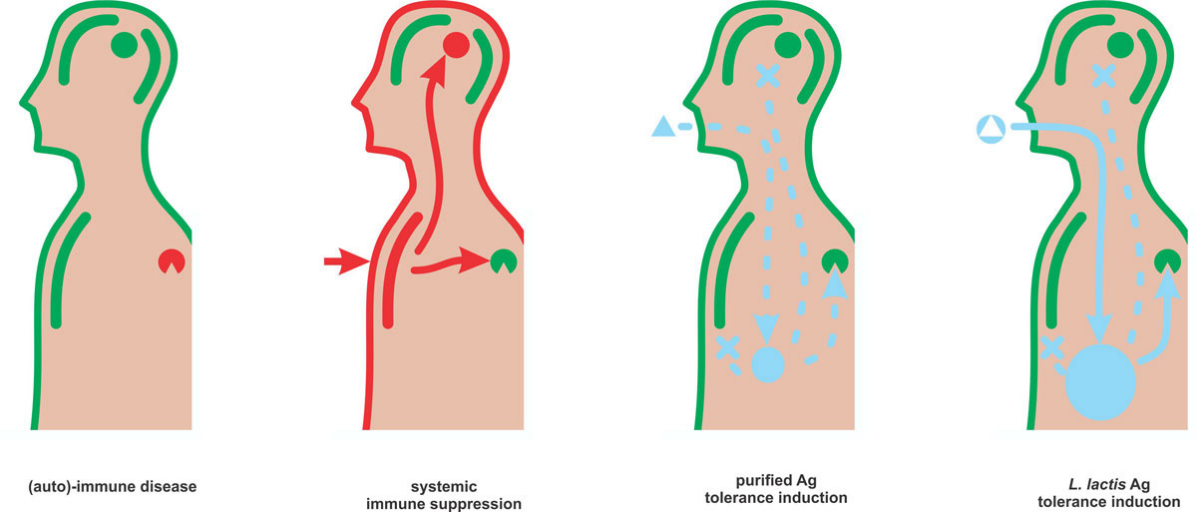
***L. lactis *can bridge the gaps towards Ag-specific immune therapy**. State of the art research on (auto-) immune diseases allows for mechanistic understanding of these pathologies, in terms of onset, precise localization (red circle), cellular compartment and specificity of the auto-Ag (Ag; wedge). The current standard of care exists of systemic immune suppression which may alleviate some aspects of the immune pathology but very often, due to its systemic and Ag-nonspecific nature, comes associated with moderate to even severe toxicity in otherwise non-affected organs. Purified Ag (blue triangle), delivered through the oral route, aims to make proficient use of inherent oral tolerance induction at the intestinal mucosal immune system to provoke Ag-specific suppression of the immune disease without affecting areas that do not share the Ag (X). This route of administration however suffers from major practical obstacles which make it difficult to administer sufficient, high quality Ag to the intestinal mucosa. To circumvent this, recombinant *L. lactis *expressing the Ag (blue circle holding white triangle) can be delivered through the oral route. These bacteria can then synthesize Ag of reproducible quality directly at the intestinal immune system and by co-synthesis of immune modulatory components can further enhance tolerance induction. In this way, Ag-specific oral tolerance induction is rendered practically feasible.

Along with the concept of using recombinant *L. lactis *for vaccination (recently reviewed [74]) arose the idea of making this host a carrier for tolerance induction (Figure 2). It is indeed not far-fetched thinking to engage an innocuous, well-known food derived host for dampening rather than stimulating immune reactions.

Initial studies in animals show alleviation of allergic responses subsequent to the administration of recombinant *L. lactis*. Mucosal - oral or intranasal - administration of recombinant *L. lactis *that secrete bovine beta-lactoglobulin (BLG) leads to the induction of BLG specific fecal IgA [75]. T helper (Th) 1 Th1 cells stimulate cellular rather than humoral immunity and antagonize Th2 cells, which often underlie allergy. Oral application of *L. lactis *secreting BLG prior to sensitization enhances Th1 response in serum (increased IgG2A, decreased IgE) and ex-vivo splenocytes (increased IFNγ). However, in some settings, splenocytes showed increased interleukin (IL)-5 (Th2). Cellular location of the Ag seems to matter: intracellular BLG seems to drive Th2 response while secreted BLG enhances both Th1 as well as Th2 features, the latter associated with a - less pronounced - IgE induction which is suggestive for reduction of sensitization. On the other hand, IgA, a desired feature for the establishment of tolerance, is induced most prominent in the setting where IgE is not reduced versus the reference [76]. When inoculated intranasal prior to sensitization, recombinant *L. lactis *producing intracellular major birch pollen allergen BetV1 decrease allergic response to BetV1. Specific serum IgE was decreased and both specific IgG2A and IgG1 are increased. Broncheoalveolar lavages show reduced IL-5 and reduced number of eosinophils, while, both in the airways as well as in the gut, specific IgA was increased [77]. Intranasal inoculation of *L. lactis *producing BLG post sensitization reduced mucosal as well as systemic IgG1 and showed diversion of splenocytes towards Th1 [78]. Taken together, these reports propose the induction of IgA and the avoidance of Th2 response as prominent endpoints and show that - at least to a certain extent - these can be met by use of recombinant *L. lactis*. The reported synthesis of other known allergens such as peanut allergen Ara h 2 [79] and buckwheat major allergenic storage protein [80] demonstrates that the repertoire of potential immune therapeutic *L. lactis *is gradually expanding and awaits experimentation.

A limited number of reports demonstrate that tolerance induction through recombinant *L. lactis *can be expanded to auto-Ags. Heat shock proteins (Hsp) form a class of proteins involved in the management of cellular stress and are drivers of inapt immune reactions and their associated pathologies. Bacterial Hsp proteins can provoke cross reactivity to their human counterparts, these then in turn becoming auto-Ags. Oral administration of *L. lactis *producing Hsp65 shows potential for immune intervention. *L. lactis *Hsp65 attenuates atherosclerosis in susceptible mice. Both secreted as well as intracellular mycobacterial Hsp65 reduced splenocyte proliferation and atherosclerotic lesions [81]. Further, *L. lactis *producing *Mycobacterium leprae *Hsp65 prevented experimental autoimmune encephalomyelitis in mice by Treg induction [82].

These studies show that, as a carrier for the delivery of immune-pathological epitopes, *L. lactis *undoubtedly holds promise for anti-allergic and autoimmune therapy: this carrier certainly does not lead to worsening of the autoimmunity or allergy. Caution however should be taken when translating to human medicine. A fundamental aspect of the above mechanisms involves the induction of Th1 responses leading to avoidance of allergic - Th2 - responses. A large portion thereof will evidently be provoked by IFNγ production, which in turn may well be a result of the challenge of naïve mice with *L. lactis*, which is not part of its diet and/or the gastrointestinal microflora. Left alone the fact that Th1/Th2 distinction may not be as clear in humans as it is in mice, nearly no humans are naïve to *L. lactis*, again due to diet composition. The case may even be more obscure for other bacterial hosts as it will be difficult if not impossible to predict which part of the population has been exposed to any said strain or bioactive component thereof. Novel therapeutic approaches should therefore carry sufficient immune modulatory information, for which exists a rationale that substantiates mouse to human translation, enabling them to act autonomous from primary immune reactions that occur upon naïve encounters.

Many probiotic strains - lactic acid bacteria and other - exert beneficial health effects through surface and secreted proteins, glycoproteins, teichoic acids and lipopolysaccharide. To provoke responses, these molecules interact with pattern recognition receptors of the host [83]. A number of reports show that also *L. lactis *strains can exert similar immune modulatory effects [78,84-86], amongst others driven by teichoic acids [87], also in conjunction with purified allergen [88]. Again, this shows that *L. lactis *in itself is a good candidate delivery vehicle. However, for the reasons described above, caution should be taken when translating these effects to use in humans. A more rational approach for this type of drug design therefore lies in applying signaling pathways derived from the immune system that have shown to be paralleled in both the experimental animal model as well as in humans. The combination of selected recombinant Ag and cytokines therefore allows for more predictable steering of pathways for tolerance induction.

The initial findings that cytokines could be delivered to the mucosa through secretion by recombinant *L. lactis *led to the development of a new type of immune modulatory *L. lactis *[6,7]. Along these lines of thinking, IL-10 secreting *L. lactis *[89,90] and *L. lactis *producing IL-12 have been used for anti-allergic therapy [13]. The latter approach provides a good alternative to influence Th1/Th2 balance, as shown in ovalbumin-induced asthma [91].

## Recombinant *L. lactis *can induce Ag-specific reduction of cellular immunity

Ag-specific tolerance induction remains the ultimate goal to treat autoimmune and allergic diseases. Oral administration of *L. lactis *delivering Ags has already demonstrated efficacy by inducing Ag-specific immune suppression. Huibregtse et al. report a detailed mechanism for T cell-driven Ag-specific tolerance induction by studying oral administration of recombinant *L. lactis *secreting ovalbumin (LL-OVA) in a mouse model carrying transgenic OVA-specific T cell receptors [92]. OVA-sensitized transgenic mice were exposed to oral LL-OVA. Subsequent, OVA-specific tolerance was assessed by delayed-type hypersensitivity measured by ear thickening. *L. lactis*-mediated intestinal delivery of OVA reduced OVA-specific hypersensitivity in this experimental mouse model. This systemic suppression could not be obtained with purified OVA underlining the added value of *L. lactis *as a mucosal carrier, especially after sensitization. *In vitro *re-stimulation of the ear-draining lymph nodes, spleen and gut-associated lymph nodes with OVA revealed a higher IL-10 production in the LL-OVA treated animals, suggesting the involvement of a Treg compartment. Further *in vitro *research showed that LL-OVA feeding suppressed OVA-specific proliferation of CD4^+ ^splenocytes and this seemed to be effectuated by induced CD4^+^CD25^- ^T cells functioning in a TGF-β-dependent way. Adoptive transfer of this CD4^+^CD25^- ^T cell compartment suppressed OVA-specific reactivity in OVA-immunized balb/c recipient mice. Taken together, this study was the first to demonstrate efficacy of recombinant *L. lactis *in inducing Ag-specific Tregs and subsequent Ag-specific tolerance in an experimental mouse model. Moreover it clearly underlines the added value of *L. lactis *mediated delivery over applying purified Ag in mucosal therapy. *L. lactis *mediated Ag delivery allows for intervention subsequent to sensitization where purified Ag only allows preventive strategies. In addition, the critical amount of OVA required was reduced by several orders of magnitude, which is, as stipulated above, a highly desirable goal.

Can this approach be used to induce Ag-specific reduction of cellular immunity in a host of wild type T cell repertoire? This appears to be the case indeed, as oral *L. lactis *secreting the deamidated DQ8 gliadin epitope (one of the drivers of celiac disease) shows a major suppression of intestinal and systemic DQ8-restricted T cell responses in NOD AB° DQ8 transgenic mice [93].

## *L. lactis*: the gateway towards a novel, Ag-specific T1D therapy

Also in the context of autoimmunity, oral administration of recombinant *L. lactis *that secrete auto-Ags shows to be a valid approach to restore tolerance. A new therapeutic strategy for T1D was devised by use of recombinant *L. lactis *secreting human pro-insulin (PINS) and IL-10 (LL-PINS+IL-10) to recently-diagnosed diabetic NOD mice [94]. A combination therapy (CT) of LL-PINS+IL-10 with a short-course of subtherapeutic doses anti-CD3 Abs reversed diabetes in 59% of treated mice in comparison to 25% with anti-CD3 monotherapy and 15% when using LL-PINS+IL-10 alone. Moreover anti-CD3 plus LL-PINS reversed diabetes in 49% of treated mice highlighting the importance of mucosal co-delivery of IL-10 and Ag. This observation underlines the great potential of combination strategies: systemic immunomodulation is combined with Ag-specificity, thereby providing specificity and increasing efficacy. As such, toxic dosages of systemic immunomodulators can be reduced to non-harmful - but as such sub-therapeutic - levels.

Successful CT was associated with preservation of remaining β-cell mass, not with increased proliferation or generation of new β-cells. Moreover, cured CT-treated animals showed increased frequencies of CD4^+^CD25^+^Foxp3^+ ^Tregs in pancreatic-draining lymph nodes. These Tregs exhibited polyclonal and Ag-specific suppressive capacity *in vitro *in addition to disease-specific suppressive capacity *in vivo*. Also, locally in the pancreas, increased frequencies of proliferating Foxp3^+ ^Tregs could be detected, further suggesting that CT of low-dose anti-CD3 with LL-PINS+IL-10 induces PINS-specific Tregs that migrate to the site of inflammation and inhibit further β-cell destruction.

To verify whether other auto-Ags can induce or enhance diabetes remission, recombinant L. lactis secreting T1D-related auto-Ag GAD65 have been tested successfully in a similar setting [95]. Interestingly, LL-GAD CT retained a similar 60% diabetes remission even in severely hyperglycemic (late) NOD mice as opposed to LL-PINS+IL-10 CT. This could be explained by the hypothesized primary role for PINS in the autoimmune cascade followed by GAD65 reactivity and suggests that LL-GAD CT is more widely applicable and can be used in more advanced stages of T1D.

## Bringing recombinant *L. lactis *to the clinic

Moving recombinant *L. lactis *forward to clinical experimentation is the next step in achieving the ultimate goal: the development of a novel pharmaceutical product. Key aspects encompass clear and unambiguous genetic engineering, undisputable safety profile and high quality chemistry, manufacturing and controls (CMC). The whole of these will be at the center of the authorities' scrutiny and precise approach will lay the foundation for regulatory approval.

The regulatory approval process to use live recombinant bacteria as drugs faces unique challenges. First, research and production operations need to be elevated to the standard of the pharmaceutical industry. According to guidelines from "International Conference on Harmonization of Technical Requirements for Registration of Pharmaceuticals for Human Use" (ICH), production of pharmaceuticals must comply with good manufacturing practice (GMP). Product quality and consistency needs to be demonstrated, including testing of identity, purity, potency and stability. Animal toxicology studies must demonstrate the safety of the drug product before it can be administered to humans. Second, drugs that are deliberately releasing recombinant organisms need to follow special guidelines addressing environmental containment and eradication. As an inherent part of communication towards society of its know-how on environmental safety, the clinical trial sponsor will need to demonstrate appropriate risk assessment and must avail of a superbly functioning containment system [96].

The bacterial organism of choice is by absolute preference well described and with a long standing history of safe use in humans. For this it should have very few if any case records in medical literature. Growth of the host should be susceptible to a panorama of antibiotics but it may be an advantage if a certain number of those will still allow for Ag expression. Residence of the organism inside the host - whether transient, lingering or capable of active colonization - will be determining the degree to which a handle on dosage and timing will exist, both key aspects of a clear pharmacodynamic profile. From this point of view, *L. lactis *has a clear advantage over other hosts. It is a well described, non-colonizing species, with an excellent track-record of safe use in humans. More specific, as described in detail above, many groups showed safety in tolerance induction in that recombinant *L. lactis *abstain of any aggravation of the studied (auto-)immune pathologies. Further, *L. lactis *is susceptible to a range of antibiotics that prevent growth but not recombinant protein expression.

Genetic engineering must be robust and should preclude the use of plasmids and other mobile elements and should be free of antibiotic selection markers. This makes the bacterial chromosome, at sites away from transposons and IS elements, the desired carrier for Ag gene expression. The genetic engineering must ensure environmental containment, eventually by a combination of inherent features of the species and by recombinant traits [96].

Following demonstration of biological activity in animals, drug development - also when starting from a recombinant bacterium - follows set rules. As for any pharmaceutical, establishing a medicinal product requires the set-up of adequate CMC. The process of genetic engineering, strain selection and maintenance of a research cell bank is straightforward and well known to most researchers in the field. These activities are - almost by definition -non-GMP processes. Documentation must nevertheless be of the highest quality because at this stage, identity of the active component is fixed and stability of the transgenic trait and containment system must be demonstrated. Every activity beyond the final strain selection - the establishment of a master cell bank, the development of the processes for fermentation and biomass concentration as well as lyophilization to produce a drug substance in bulk and stability studies - need to comply with GMP standards and documentation should be recorded accordingly. All segments of the production process must be robust - i.e. should not overly depend on any variable - and scalable. All raw materials have to be of clinical grade and by high preference, if not exclusively, free of all animal-derived products. The product must be safe and therefore the very initial development phase will encompass toxicology studies in relevant animal species and small, phase I safety and tolerability studies in humans. The drug must be sufficiently stable to allow treatment of thousands of patients. At any of the relevant stages during processing, thorough and reliable, validated documentation is required.

Approval or rejection of an application to perform a clinical trial resides with national or European agencies. Initially, all regulatory systems afford drug developers a certain period of scientific advice during which they can be approached to discuss future steps of product development. Such a consultancy phase allows the listing of supporting studies the agencies consider necessary, including additional animal proof of concept and safety studies. Without this window of initial communication before the filing of the final product, a late discovery of gaps in the data in a more advanced phase could derail an entire project.

Although all clinical trial applications require data on animal pharmacology and toxicology, manufacturing information, clinical protocols and investigator information, regulatory agencies in different countries may have unique requirements which need to be taken into account when setting up a dossier. When comparing Europe vs. North America the contents of the application may vary substantially, mainly differing in whether all or part of the existing documentation must be included. U.S. authorities (U.S. Food and Drug Administration; FDA) require having all of the documentation in the file. European authorities (European Medicines Agency; EMA) request an overview on strategy and a product description, but supporting data remain at the sponsor, eventually to be presented upon request. Canadian authorities (Health Canada) have very concise regulations, and will request all documentation to be kept at the study sites, again to be presented upon request. Where most authorities will require clinical trial applications (CTA) per specific trial; U.S. FDA utilizes the "open" investigational new drug (IND) process. The IND is product specific and the sponsor continues to submit new data along the road towards market approval.

Any clinical trial involving recombinant organisms will be categorized as "contained use" or "deliberate release", the first requiring absolute physical containment, the latter harmonizing the use of the recombinant organism and its release in the environment. For most authorities the application for contained use - provided physical containment is absolute, which is left at the discretion of the sponsor - is relatively straightforward. Medical use however is almost inherently coupled to deliberate release of the recombinant organisms in the environment. Regulations on the deliberate release of recombinant organisms differ between countries, and the application process involves differing steps. The U.S. system sets in place the NIH Recombinant DNA Advisory Committee (RAC) to advise local authorities in their decisions when overseeing a clinical trial application involving recombinant organisms. Decisions on the use of recombinant organisms however remain with the clinical centers. Canadian and European authorities will decide on the use of recombinant organisms on a national level. Canadian authorities apply no guidelines on the use of recombinant organisms. The Canadian environmental protection agency however will judge any novel product - regardless of its origin - to be entered into Canada on its potential environmental impact upon "deliberate" release. The "new substance notification" application undergoes thorough scrutiny, in the specific case of recombinant microflora on data to demonstrate the effectiveness of the containment system and experiments and literature that allow judgment on environmental impact. Specific for the European review and approval process is the scrutiny for products involving recombinant DNA, which is regarded as fundamentally different from conventional approaches.

The above, complex path is essential to guarantee safety to humans. When entering into human tolerance induction therapy, this path is however not expected to differ extensively from that of earlier clinical trials with recombinant *L. lactis *that have been conducted successfully (http://ClinicalTrials.gov Identifiers NCT00729872 and NCT00938080 [15]). Therefore, the conduct of clinical trials using recombinant *L. lactis *for Ag-specific tolerance induction has moved beyond the visionary to become practicable.

## Conclusion: *L. lactis *can bridge the gaps towards Ag-specific immune therapy

Immune diseases driven by innocuous - auto or allo - Ags are rapidly emerging. As judged from their global evolution, this phenomenon goes along with western lifestyle [97,98]. The manifestations of these diseases come in a wide variety, from "unpleasant" to "life threatening when left untreated". Despite our growing mechanistic understanding, the current standard of care remains unsatisfactory in many cases. Shortcomings range from partial and temporal relief to being associated with highly undesirable, widespread toxic side effects. It will be necessary to harness our molecular immunological understanding in order to develop medication that can reach the suitable outcome: Ag-specific tolerance induction devoid of side effects. Oral tolerance, a system that manages the discrimination between friend and foe in the intestine, is one possible entry into this field. However it remains largely untapped because of practical and technological hurdles. In prophylactic settings, clear demonstration of Ag-specific oral tolerance induction has been given in experimental animals. It however remains very difficult to deliver sufficient amounts of high quality Ag to the human intestine. Rather than being merely preventive, *L. lactis *Ag-delivery has shown suitable to support intervention strategies in Ag-driven immune diseases. With recombinant *L. lactis *we avail of the technological tools to bridge between our molecular understanding and the practicalities of human medicine. We can engineer *L. lactis *to produce (auto-) Ags and immune modulatory factors and genetic engineering is such that it is safe to use. Large scale GMP manufacturing has been established and quality controls, both critical components for clinical experimentation under ICH guidelines, are in place. All this has been shown necessary and sufficiently elaborated to allow successful completion of the regulatory approval process. We now find ourselves at a pivotal point: we have the tools and experience to allow recombinant *L. lactis *to make a difference in healthcare by helping out people suffering from prominent but largely unmet medical needs.

## Competing interests

L.S. is co-founder, shareholder and employee of ActoGeniX NV.
